# Newspaper Enterprise

**Published:** 1902-12

**Authors:** 


### NEWSPAPER ENTERPRISE.

THE *Buffalo Express,* on Tuesday, October 14, presented its readers with an interview with Dr. Florestan Aguilar, of Madrid, Spain, a "dentist of international fame," who "has been in this city for a few days, the guest of Dr. William C. Barrett, of the University of Buffalo."

The article is introduced by a history of Dr. Aguilar and his work and the honors bestowed upon him in Spain, and then it gives the doctor an opportunity to tell what he knew of the feeling of his people in regard to the Spanish-American war.

"You won the battle of arms," said he, "but we won the diplomatic battle, and had the best of the bargain. You paid us $20,000,000 for the Philippines. They were of no use to us. . . . As for Cuba, its loss, while humiliating to our national pride, has been really a benefit to us, for there was no revenue from it that could compensate for the annoyance. . . . Spaniards who settled in that country have, since its loss, returned to Spain and brought with them the fortunes accumulated there. . . . Hence my country is resigned," etc. Thus through a column this interview continues. And it is unnecessary to add that it created marked comment from the press of New York State.

The most remarkable part of this extraordinary interview was, however, omitted, and that was that Dr. Aguilar left Buffalo for his return passage September 27, just eighteen days prior to the time that the interview took place, October 14, and on October 10 he was in Paris attending the Executive Council of the Fédération Dentaire Internationale, and made his report to that body of his mission to the United States with which he had been intrusted.

That this interview is full of interest there can be no question, and were it not for the slight mixing up of dates it would be entirely satisfactory, even to Dr. Aguilar, as it, doubtless, represents his views on the Spanish-American War, but while this is true, there is always a certain number of curious people who would like to know how such an interview was procured, whether by cable, wireless telegraphy, telepathy, or whether it is a new effort on the part of the daily press to convey truth in the form of a novelette. In view of all the facts, it will certainly be entertaining reading for Dr. Aguilar in his far-away Madrid home, and will give him some idea of the perfection to which interviewing has been reached in the United States.

